# Proteome characteristics of liver tissue from patients with parenteral nutrition-associated liver disease

**DOI:** 10.1186/s12986-020-00453-z

**Published:** 2020-06-03

**Authors:** Gulisudumu Maitiabola, Feng Tian, Haifeng Sun, Li Zhang, Xuejin Gao, Bin Xue, Xinying Wang

**Affiliations:** 1 Department of General Surgery, Jinling Hospital, Medical School of Nanjing University, East Zhongshan Road 305, Nanjing, 210002 P.R. China; 2 Core Laboratory, Sir Run Run Hospital, Nanjing Medical University, Nanjing, 211166 China

**Keywords:** Parenteral nutrition associated liver disease, Mitochondria, Oxidative phosphorylation, Metabolic disorder, Oxidative stress

## Abstract

**Background:**

Parenteral nutrition (PN)-associated liver disease (PNALD) is a common and life-threatening complication in patients receiving PN. However, its definitive etiology is not yet clear. Therefore, performed proteomic analyses of human liver tissue to explore the same.

**Methods:**

Liver tissue was derived and compared across selected patients with (*n* = 3) /without (*n* = 4) PNALD via isobaric Tag for Relative and Absolute Quantitation (iTRAQ)-based quantitative proteomics. Bioinformatics analysis was performed using Gene Ontology (GO) and Kyoto Encyclopedia of Genes and Genomes (KEGG) databases to explore the mechanisms of PNALD based on differentially expressed proteins (DEPs). The essential proteins that were differentially expressed between the two groups were explored and verified by western blotting.

**Results:**

A total of 112 proteins were found to be differentially expressed, of which 73 were downregulated, and 39 were upregulated in the PNALD group. Bioinformatics analysis showed DEPs to be associated with mitochondrial oxidative phosphorylation (mainly involved in mitochondrial respiratory chain complex I assembly), hepatic glycolipid metabolism (involved primarily in glycogen formation and gluconeogenesis), and oxidative stress (mainly involved in antioxidant change).

**Conclusion:**

Overall, our results indicated that mitochondrial energy metabolism impairment, hepatic glycolipid metabolism disorder, and excessive oxidative stress injury might explain the comprehensive mechanism underlying PNALD. Moreover, we have provided multiple potential targets for further exploring the PNALD mechanism.

## Background

Total parenteral nutrition (TPN) is a vital therapeutic measure for patients with impaired gut function, including short bowel syndrome, severe inflammatory bowel disease, or chronic idiopathic intestinal pseudo-obstruction [1–3]. Parenteral nutrition-associated liver disease (PNALD) is one of the most common complications of long-term parenteral nutrition (PN), which severely impairs the physical health of patients, and is the primary factor limiting long-term PN therapy.

The incidence of PNALD in infants receiving long-term parenteral nutrition is between 25 and 60%, and that in adults is between 15 and 40% [4]. In adults, a history of PNALD is characterized by elevated liver enzymes and associated steatosis, with ensuing complications such as steatohepatitis, cholestatic hepatitis, as well as fibrosis and cirrhosis [5]. Most biochemical abnormalities of the liver can be reversed by weaning at an early stage of PNALD, whereas most of the advanced pathophysiological changes are irreversible, resulting in cirrhosis, decompensated liver disease, liver failure, and liver carcinoma. In these patients, PNALD progresses to end-stage liver disease, requiring combined intestinal and liver transplant [6–8].

Research on the mechanistic pathways and ameliorative modalities in PNALD is a significant focus in the field of gastroenterology and hepatology. Several theories have been proposed, for instance,the components of PN directly harm the liver or that the absence of enteral nutrition disrupts the enterohepatic axis leading to liver injur y[9]. However, the pathophysiology and etiology of PNALD remain unclear, and there have been very few studies exploring the overall protein expression in liver tissue of patients with PNALD.

Proteomics is a large-scale comprehensive study of proteins, including information on protein abundance and modification, along with their interacting networks [10]. Studies regarding PNALD have been limited to the transcriptomic level in mouse models [11], and, to the best of our knowledge, neither genomic nor proteomics studies on PNALD have been conducted till date. More importantly, no prior studies have explored the use of human specimens to understand this disease. Studies are, thus, required that use liver samples from PNALD patients for proteomics research, which would be more conducive to solving clinical problems. Based on this consideration, this study aimed to explore the possible mechanisms underlying the pathogenesis of PNALD, as well as identify potential therapeutic targets by performing hepatic proteomics in patients with or without PNALD in the present study.

## Materials and methods

### Subjects

Patients with diagnostic percutaneous or intraoperative liver biopsy were recruited from the Department of General Surgery, Jinling Hospital, Medical School of Nanjing University. Liver tissue was obtained from seven patients, with (*n* = 3) and without (*n* = 4) PNALD. Disease information and clinical characteristics were collected from the electronic database and detailed in Table 1. Subjects with PNALD had liver enzymes elevated 1.5 times the upper limit of normal, in the absence of any other cause, such as viral hepatitis or drug-induced changes [12]. All subjects gave their informed consent for inclusion before they participated in the study. The study was approved by the Ethics Committee of the Jinling hospital, Medical School of Nanjing University (2017NZGKJ-071).

**Table 1:** Demographic and Clinical Characteristics of Participants

Group	PNALD			Non-PNALD				
Characteristic	Pt. 1	Pt. 2	Pt. 3	Pt. (A)	Pt. (B)	Pt. (C)	Pt. (D)	P-value
Age (yr.)	58	49	31	46	52	62	38	0.5545
Sex	man	man	man	man	man	Woman	Woman	–
Weight (kg)	60	61	43	72	76	60	52	0.2604
BMI, kg/m2	21.6	20.6	15.8	23.2	24.5	23.1	20.1	0.1277
Diagnosis	short bowel syndrome	short bowel syndrome	abdominal cocoon	hepatic hemangioma	Cholelithiasis	hepatic hemangioma	hepatic hemangioma	–
ALT (U/L)	136	129	217	22	32	34	31	0.0027
AST (U/L)	132	125	257	26	28	21	29	0.0099
GGT (U/L)	335	281	458	39	41	36	27	0.0007
TBIL (umol/L)	60.7	45.4	45.5	6.9	10.2	8.3	7.7	0.0003
BUN (mmol/L)	8.9	14.1	9.5	3.4	4.6	3.9	5.2	0.0063
Scr (umol/L)	50	189.8	68	79	83	69	77	0.5187
Days with PN	123	109	138	0	0	0	0	–

*ALT* aspartate aminotransferase, *AST* alanine aminotransferase, *GGT* gamma-glutamyl transferase, *TBIL* total bilirubin, *BUN* blood urea nitrogen, *Scr* serum creatinine, *Pt* patient

### Sample preparation

The tissues were ground in liquid nitrogen. One milliliter of lysis buffer (7 M urea, 4% SDS, 1x Protease Inhibitor Cocktail (Roche Ltd. Basel, Switzerland)) was added to samples, followed by sonication on ice and centrifugation at 13000 rpm for 10 min at 4 °C. The supernatant was transferred to a fresh tube*.*

### Protein digestion and iTRAQ labeling

Determine the protein concentration of the supernatant using the BCA protein assay, and then transfer 100 μg protein per condition into a new tube and adjust to a final volume of 100 μL with 100 mM TEAB (triethylammonium bicarbonate). Add 5 μL of the 200 mM DTT and incubate sample at 55 °C for 1 h, then add 5 μL of the 375 mM iodoacetamide to the sample and incubate for 30 min protected from light at room temperature. For each sample, proteins were precipitated with ice-cold acetone, and then were redissolved in 20 μL TEAB. Then proteins were tryptically digested with sequence-grade modified trypsin (Promega, Madison, WI), and the resultant peptide mixture was labeled using chemicals from the iTRAQ reagent kit. The labeled samples were combined, desalted using C18 SPE column (Sep-Pak C18, Waters, Milford, MA) and dried in vacuo.

### High-pH reverse-phase separation

The peptide mixture was re-dissolved in buffer A (buffer A: 10 mM ammonium formate in water, pH 10.0, adjusted with ammonium hydroxide), and fractionated by high-pH separation using an Acquity UPLC system (Waters Corporation, Milford, MA) connected to a reverse-phase column (BEH C18 column, 2.1 mm × 150 mm, 1.7 μm, 300 Å, Waters Corporation, Milford, MA). The high-pH separation was performed using a linear gradient, starting from 0% B to 45% B in 45 min (B: 10 mM ammonium formate in 90% ACN, pH 10.0, adjusted with ammonium hydroxide). The column flow rate was maintained at 250 μL/min, and the column temperature was maintained at 45 °C. Twelve fractions were collected, and each was dried in a vacuum concentrator for the next step.

### Low-pH nano-HPLC-MS/MS analysis

Fractions were re-suspended with 32 μl solvent C (C: water with 0.1% formic acid; D: ACN with 0.1% formic acid), separated by nanoLC, and analyzed by on-line electrospray tandem mass spectrometry. Experiments were performed on a nanoACQUITY UPLC system (Waters Corporation, Milford, MA) connected to a quadrupole-Orbitrap mass spectrometer (Q-Exactive) (Thermo Fisher Scientific, Bremen, Germany) equipped with an online nano-electrospray ion source. Eight-microliter peptide sample was loaded onto the trap column (Thermo Fisher Scientific Acclaim PepMap C18, 100 μm × 2 cm), with a flow rate of 10 μl/min for 3 min, and subsequently separated on the analytical column (Acclaim PepMap C18, 75 μm × 25 cm) with a linear gradient, from 5% D to 30% D in 105 min. The column was cleaned and re-equilibrated to initial conditions for 5 min. The column flow rate was maintained at 300 nL/min, and the column temperature was maintained at 45 °C. The electrospray voltage of 1.8 kV versus the inlet of the mass spectrometer was used.

The Q-Exactive mass spectrometer was operated in the data-dependent mode to switch automatically between MS and MS/MS acquisition. Survey full-scan MS spectra (m/z 350–1600) were acquired with a mass resolution of 70 K, followed by fifteen sequential high-energy collisional dissociations (HCD)-MS/MS scans with a resolution of 17.5 K. In all cases, one Microscan was recorded using a dynamic exclusion of 30 s. MS/MS-fixed first mass was set at 100.

### Database searching and data analysis

Tandem mass spectra were extracted by Proteome Discoverer software (Thermo Fisher Scientific, version 1.4.0.288). Charge state deconvolution and deisotoping were not performed. All MS/MS samples were analyzed using Mascot (Matrix Science, London, UK; version 2.3). Mascot was set up to search the Uniprot-SwissProt database (Taxonomy: *Homo sapiens*, 20,245 entries) assuming the digestion enzyme trypsin. Mascot was searched with a fragment ion mass tolerance of 0.050 Da and a parent ion tolerance of 10.0 PPM. Carbamidomethyl of cysteine and iTRAQ 8plex of lysine and the n-terminus were specified in Mascot as fixed modifications. Oxidation of methionine and iTRAQ 8plex of tyrosine were specified in Mascot as a variable modification. Use the percolator algorithm to control peptide level false discovery rates (FDR) lower than 1%. Only unique peptides were used for protein quantification, and the method of normalization on protein median was used to correct experimental bias, the minimum number of proteins that must be observed to allow was set to 1000. Bioinformatics analysis of the identified proteins was performed, and DEPs were defined in the iTRAQ experiment according to the following criteria: unique peptides ≥1, *P*-value < 0.05, fold change > 1.2 or < 0.8333 [13]. DEPs were entered into the DAVID (Database for Annotation, Visualization, and Integrated Discovery) database (david.abcc.ncifcrf.gov) for functional classification and GO enrichment analysis [14], and to determine the significant pathways according to the KEGG pathway analysis (www.kegg.jp/kegg/pathway.html). Protein-protein interaction (PPI) networks were generated through the STRING database (v10, string-db.org). Pathway analysis was conducted using Ingenuity Pathway Analysis (IPA) (www.biotree.com.cn Shanghai Biotree biotech Co., Ltd) on the differentially expressed proteins.

### Verification of proteins by western blot analysis

Protein samples were separated by electrophoresis on 12% SDS-PAGE gel, transferred to polyvinylidene fluoride membranes, blocked with 5% non-fat milk for 1 h, and incubated with primary antibodies against CYP2B6, DDAH1 (Thermo Scientific, Rockford, IL), FABP5 (ProteinTech, Chicago, IL, USA), CAPG, and NDUFA1 (Affinity Bioscience, USA), overnight at 4 °C. Anti-rabbit horseradish peroxidase (HRP)-conjugated antibody was used as a secondary antibody, followed by ECL substrate (Thermo Scientific, Rockford, IL) incubation, and image visualization using Tanon 5200 imaging system (Tanon, China). Gray-scale analysis of the bands was performed using ImageJ software. Data are presented as ratios of the target protein to the internal control.

*Statistical analyses -* Proteins with *p*-value < 0.05 and Fold Change < 0.83 or > 1.2 were considered as differentially expressed proteins between the two groups. Student’s t-test was used for comparison of the difference between groups.

## Results

### Comprehensive identification of proteome in human liver tissue

A total of 14,307 peptides and 3337 proteins were identified by iTRAQ analysis. Mass spectrometry results showed 112 differentially expressed proteins (DEPs), of which 73 were down regulated, and 39 were upregulated in the PNALD group (**Table S****1**).

**Fig. 1:**
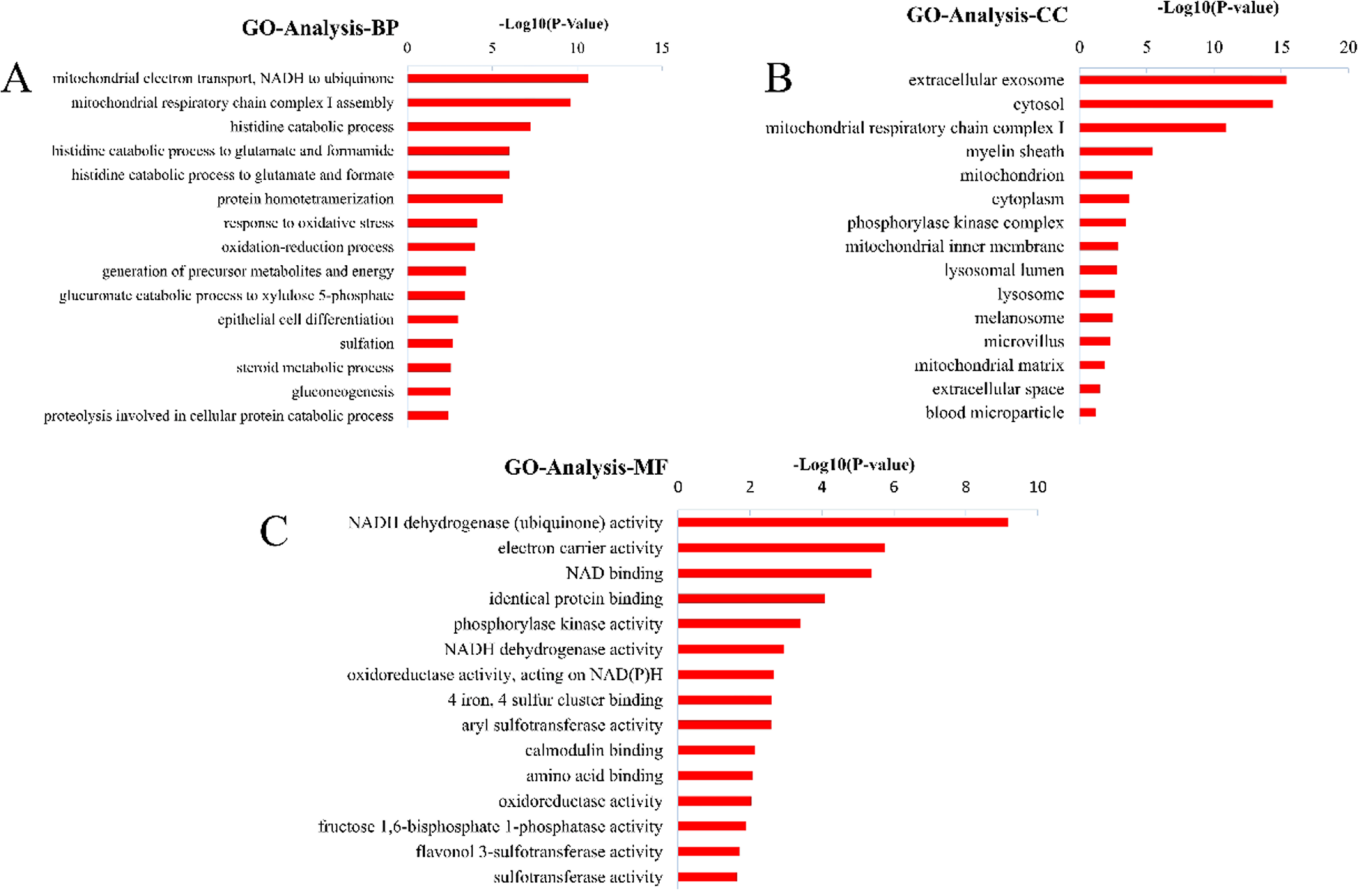
Significant Gene Ontology analysis of differentially expressed proteins. (BP, biological process; CC, Cellular component; MF, molecular function)

**Fig. 2:**
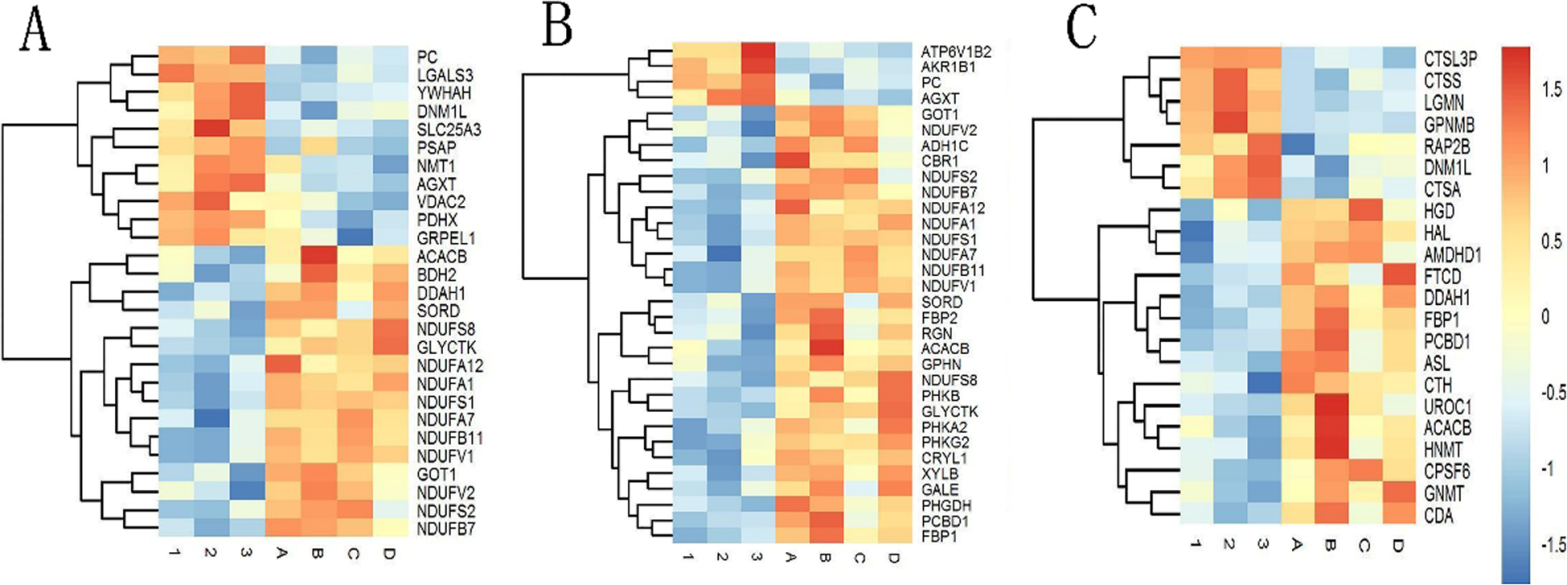
Heat map of differentially expressed proteins (DEPs) in PNALD and control patient. **a**: Mitochondrial and mitochondrial-related proteins; **b**: Glycolipid metabolisms associated proteins; **c**: Amino acid metabolisms associated proteins. Colored boxes represent upregulation (red) and downregulation (blue) in the PNALD. The color scale shown at the upper right indicates the fold changes in protein expression of all the samples. 1, 2, 3: PNALD group; A, B, C, D: Control group

To identify the functional classification of DEPs, this study performed gene ontology analysis according to their molecular functions (MF), biological processes (BP), and cellular components (CC) with the assistance of DAVID Bioinformatics Resources. The top 15 annotations represented in each of the three GO categories are shown in Fig. 1. Majority of enriched categories included mitochondrial and mitochondria-related proteins (Fig. 2a, **Table S****2**; total 27, 16 downregulated and 11 upregulated). Impaired oxidative phosphorylation was the predominant process in PNALD group. Mitochondria are known to be mainly responsible for the oxidative decomposition of dextrose, fat, and protein, for providing energy. Accordingly, the glycolipid metabolism (Fig. 2b, **Table S****3**; total 32, 28 downregulated and 4 upregulated) and amino acid metabolism (Fig. 2c, **Table S****4**; total 22, 15 downregulated and 7 upregulated) were significantly altered between the groups.

### Mitochondrial oxidative phosphorylation was impaired in PNALD patients

**Fig. 3:**
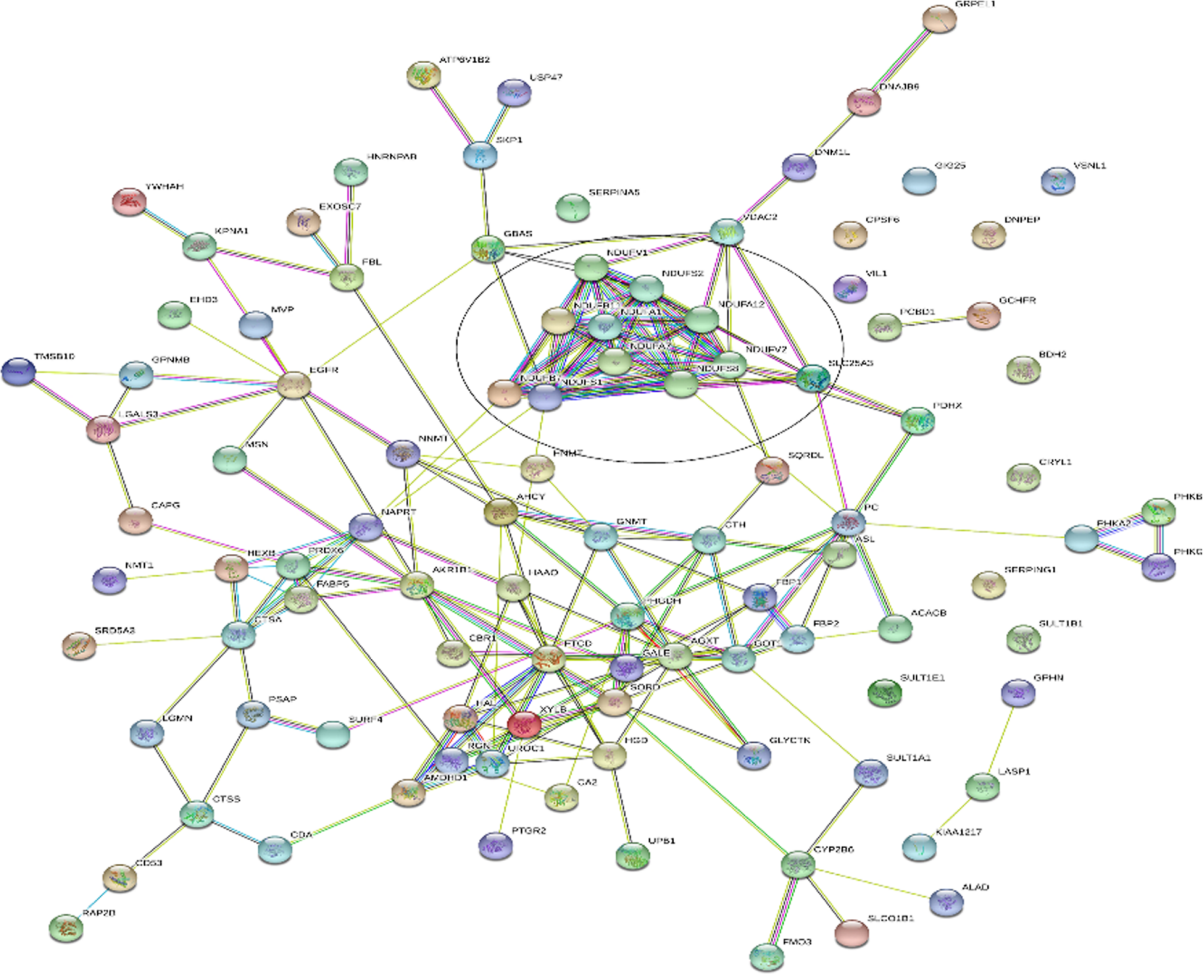
Protein-protein interaction of differentially-expressed protein for group PNALD vs. control

**Fig. 4:**
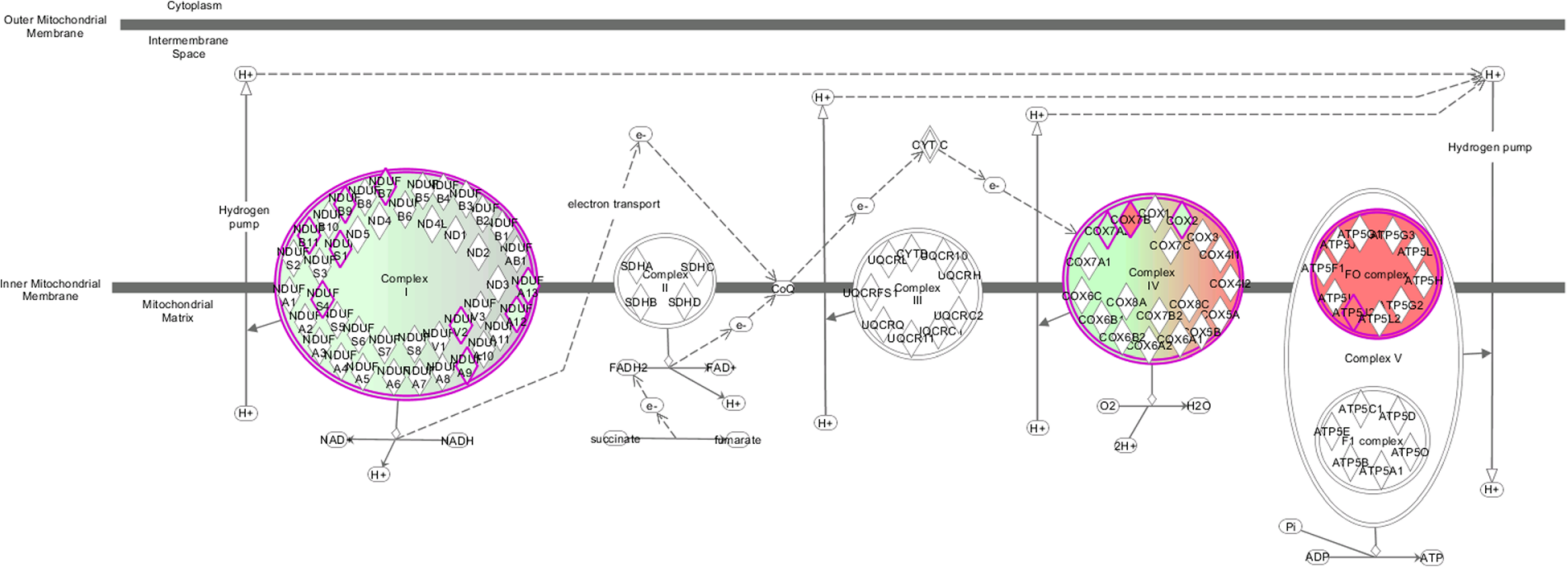
Canonical Pathway diagram from Ingenuity Pathway Analysis: Oxidative phosphorylation was significantly inhibited. Colored boxes represent upregulation (red) and downregulation (green) in the PNALD

Mitochondrial oxidative phosphorylation system is the final biochemical pathway to produce ATP and the maintenance of cell function. Mitochondrial respiratory chain NADH dehydrogenase (complex I) is the most abundant enzyme in the electron transport chain [15], and is essential for oxidative phosphorylation in mitochondria [16]. Ten subunits of NADH dehydrogenase were found to be downregulated in the PNALD group, including NDUFB11, NDUFB7, NDUFV1, NDUFA7, NDUFV2, NDUFS8, NDUFA1, NDUFS2, NDUFA12, and NDUFS1 (**Table S****2**). Consistent with our hypothesis that mitochondrial oxidative phosphorylation in the liver of patients with PNALD might be impaired, bioinformatics analysis indicated the DEPs to be enriched in mitochondria-associated biological processes (Fig. 3), including mitochondrial electron transport (NADH to ubiquinone), response to oxidative stress, oxidation-reduction process, and ATP synthesis-coupled electron transport (Table 2). And the IPA showed that the oxidative phosphorylation was significantly inhibited **(**Fig. 4**)**. These all results indicated the mitochondrial oxidative phosphorylationwas impaired in PNALD patients.

**Table. 2:** Gene Ontology analysis of mitochondria associated differentially expressed proteins

Analysis	Pathway	Protein number		
		List	Up	Down
Cellular Component	mitochondrion	NDUFB11, GRPEL1, DNM1L, NDUFB7, PSAP, NDUFA7, ACACB, VDAC2, NDUFA1, GLYCTK, NMT1, YWHAH, GOT1, NDUFV2, NDUFS8, SLC25A3, BDH2, DDAH1, NDUFS2, NDUFS1, PC	8	13
	mitochondrial respiratory chain complex I	NDUFB11, NDUFB7, NDUFV1, NDUFA7, NDUFV2, NDUFS8, NDUFA1, NDUFS2, NDUFA12, NDUFS1	0	10
	mitochondrial inner membrane	NDUFB11, LGALS3, NDUFB7, NDUFV1, NDUFA7, NDUFV2, SLC25A3, VDAC2, NDUFA1, NDUFA12	3	7
	mitochondrial matrix	GRPEL1, NDUFS8, PDHX, AGXT, NDUFS2, NDUFS1, PC	4	3
	mitochondrial outer membrane	DNM1L, ACACB, VDAC2	2	1
	mitochondrial intermembrane space	NDUFB7, NDUFS1	0	2
Molecular function	NADH dehydrogenase (ubiquinone) activity	NDUFB7, NDUFV1, NDUFA7, NDUFV2, NDUFS8, NDUFA1, NDUFS2, NDUFA12, NDUFS1	0	9
	electron carrier activity	NDUFV2, AKR1B1, PHGDH, HAAO, SH3BGRL3, NDUFS2, NDUFA12, NDUFS1	2	6
	NAD binding	AHCY, SORD, NDUFV1, PHGDH, BDH2, NDUFS2	0	6
	NADH dehydrogenase activity	NDUFV1, NDUFS8, NDUFS2	0	3
	oxidoreductase activity, acting on NAD(P)H	NDUFS8, NDUFS2, NDUFS1	0	3
	oxidoreductase activity	PTGR2, SORD, NDUFV2, AKR1B1, ADH1C, BDH2	0	6
	oxidoreductase activity, acting on the CH-CH group of donors, NAD or NADP as acceptor	SRD5A3, BDH2	0	2
Biological process	mitochondrial electron transport, NADH to ubiquinone	NDUFB11, NDUFB7, NDUFV1, NDUFA7, NDUFV2, NDUFS8, NDUFA1, NDUFS2, NDUFA12, NDUFS1	0	10
	mitochondrial respiratory chain complex I assembly	NDUFB11, NDUFB7, NDUFV1, NDUFA7, NDUFV2, NDUFS8, NDUFA1, NDUFS2, NDUFA12, NDUFS1	0	10
	response to oxidative stress	EGFR, ALAD, ATOX1, NDUFS8, NDUFS2, NDUFA12, NAPRT	1	6
	oxidation-reduction process	PTGR2, SORD, CYP2B6, PCBD1, HGD, CRYL1, CBR1, PRDX6, AKR1B1, FMO3, SRD5A3, HAAO, PHGDH, SH3BGRL3	3	11
	steroid metabolic process	CYP2B6, SULT1B1, SULT1A1, SULT1E1	0	4
	ATP synthesis coupled electron transport	NDUFA7, NDUFS1	0	2

### Hepatic glycolipid metabolism disorder in patients with PNALD

Liver plays a significant role in the control of glucose homeostasis by regulating various pathways in glucose metabolism, including glycogenesis, glycogenolysis, glycolysis, and gluconeogenesis. In this study, we observed a strong enrichment of DEPs linked to metabolic enzymes. For example, phosphorylase kinases (PHKA2, PHKB, and PHKG2), which stimulate glycogen degradation [17], were downregulated. Fructose 1,6-bisphosphate 1-phosphatases (FBP1 and FBP2), the rate-limiting enzyme in gluconeogenesis [18], were downregulated. Pyruvate carboxylase (PC), which catalyzes the ATP-dependent carboxylation of pyruvate to oxaloacetate, and involved in gluconeogenesis [19, 20], was also found to be downregulated. Consistent with the function of DEPs, bioinformatics analysis indicated that glycogen metabolic process (PHKA2, PHKB, PHKG2, and GNMT) and gluconeogenesis (GOT1, FBP1, FBP2, and PC) might be damaged in PNALD. Besides, we also found some glycolipid metabolism-associated DEPs, including nicotinamide N-methyltransferase (NNMT, upregulated) [21], D-3-phosphoglycerate dehydrogenase (PHGDH, downregulated) [22], and serine/threonine-protein phosphatase (CPPED1, downregulated) [23].

Besides its role in glucose metabolism, the liver plays a pivotal role in lipid metabolism and is the hub of fatty acid metabolism and lipid circulation [24]. Estrogen sulfotransferase (SULT1E1), involved in the process of adipogenesis [25, 26], was found to be downregulated and acetyl-CoA carboxylase 2 (ACACB), which inhibits fatty acid oxidation [27], was also downregulated. Further, lipid metabolism associated DEPs, including glycine N-methyltransferase (GNMT, downregulated) [28–32], fatty acid-binding protein (FABP5, upregulated) [33], cathepsin S (CTSS, upregulated) [34], aldose reductase (AKR1B1, upregulated) [35], and N(G), N(G)-dimethylarginine dimethylaminohydrolase 1 (DDAH1, downregulated) were also identified [36]. These results together indicated PNALD to be related to glycolipid metabolism disorder.

### Oxidative stress caused by downregulation of the antioxidant factors may generate PNALD

Previous reports had shown oxidative stress injury to be one of the significant causes of PNALD. Production of ROS is increased when mitochondrial oxidative phosphorylation is impaired and under circumstances of antioxidant defense deficiency [37]. First, the impaired mitochondrial respiratory chain complex I assembly proteins damage the normal process of oxidative phosphorylation in patients with PNALD. Second, various antioxidant factors are downregulated in PNALD. For example, peroxiredoxin-6 (PRDX6, downregulated) is a peroxiredoxin that primarily functions as an antioxidant to scavenge peroxides in biological systems [38], regucalcin (RGN, downregulated) is an antioxidant [39], delta-aminolevulinic acid dehydratase (ALAD, downregulated) is an important antioxidant enzyme, whose inhibition may result in the accumulation of its substrate d-ALA, which in turn is associated with the overproduction of ROS [40], inhibition of PHGDH (downregulated) impairs the synthesis of heme, resulting in the impairment of oxidative phosphorylation and escape of electrons to molecular oxygen generating more ROS [41], inhibition of aspartate aminotransferase (GOT1, downregulated) inhibits the synthesis of the NADPH antioxidan t[42], and DDAH1 (downregulated) deficiency significantly enhances cellular oxidative stress [43]. , Third, we found histidine metabolism-associated proteins (AMDHD1, HNMT, FTCD, HAL, and UROC1) to be downregulated in PNALD. Previous studies had reported the histidine supplementation to inhibit oxidative stress and preserve mitochondrial membrane potential as well as dehydrogenase activity [44]. Moreover, IPA results showed that the “NRF2-mediated oxidative stress response”, “EIF2 signaling”, “PFKFB4 signaling pathway”, and “Glutathione mediated detoxification” were suppressed (**Fig. S****2**). Previous studies had reported these pathways all participated in decreasing oxidative stres s[45–48]. Combined with the above results, we can hypothesize that oxidative stress caused by downregulation of the antioxidant factors may also participate in the development of PNALD.

### Validation of differential expression proteins by western blotting

**Fig. 5:**
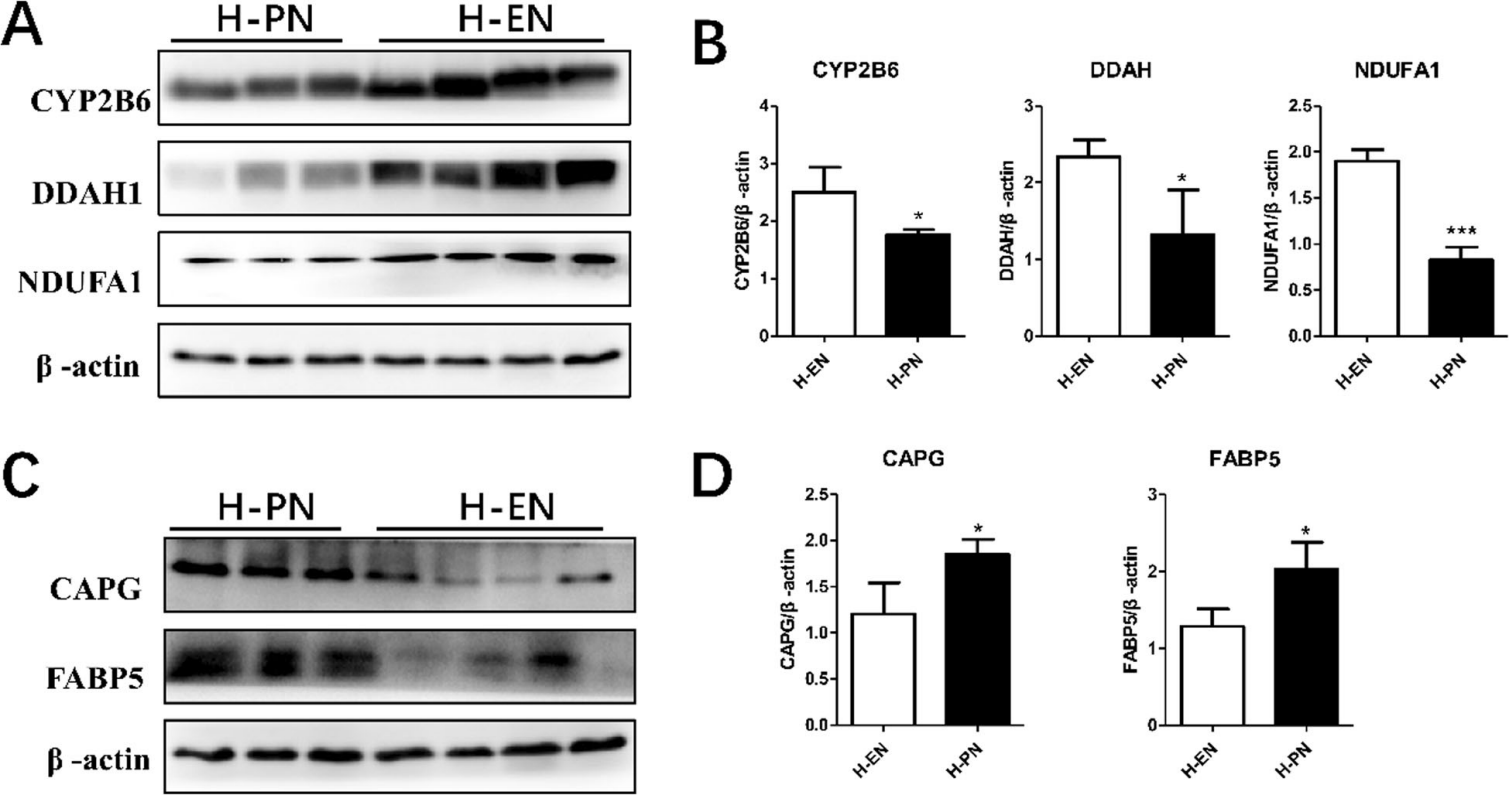
Confirmation of differentially expressed proteins (DEPs) by western blotting; **a**, **c**: Immunoblotting analysis of DEPs (CYP2B6, DDAH1, NDUFA1, CAPG, and FABP5) in PNALD(H-PN) and control (H-EN). **b**, **d**: Gray-Level scores. The β-actin protein was used as a control. *, Compared with the control group, *P* < 0.05; ***, Compared with the control group, *P* < 0.001

In order to verify the credibility of proteomics, we randomly selected several up- and downregulated DEPs for semi-quantitative verification by western blotting, including CYP2B6, DDAH1, NDUFA1, FABP5, and CAPG (Fig. 5). As shown, expression of CYP2B6, DDAH1, and NDUFA1 was significantly downregulated, and that of FABP5 and CAPG was upregulated in the PNALD group, compared to the control group. Western blotting results were in agreement with iTRAQ proteomics results.

## Discussion

Total parenteral nutrition is a life-saving therapy for patients with chronic gastrointestinal failure [49]. However, related metabolic complications, especially PNALD, arise with prolonged PN [50]. This study represents the first-ever investigation of liver tissue proteomic profiles of patients with PNALD to identify potential biomarkers and elucidate the molecular alterations that occur following PNALD. We successfully identified 112 DEPs between PNALD and control groups. The number and interaction of DEPs indicated the mitochondrial oxidative phosphorylation damage, glycolipid metabolism disorder, and oxidative stress injury as primary biological changes occurring in PNALD. In addition, we performed western blot analysis and found the results matching perfectly with the iTRAQ proteomics data.

In our study, the cellular localization of DEPs (predicted using GO analysis) revealed most of the identified putative proteins to be localized in mitochondria, which may explain the disorders in glucose and lipid metabolism. Mitochondria are best known for harboring pathways involved in ATP synthesis through the tricarboxylic acid cycle and oxidative phosphorylation [51]. More than 80% of ATP is produced by mitochondrial oxidative phosphorylation. An earlier study had reported that TPN deteriorates hepatic mitochondrial function [52]. Consistent with this, our current study indicated that multiple subunits or components of the respiratory chain complex I assembly proteins, which are essential for oxidative phosphorylation in mitochondria [16], are downregulated in PNALD. This may affect the biosynthesis of ATP, which is necessary for the maintenance of liver function. Mean ATP levels in PN mouse model were found to be reduced as compared to those in an EN mouse, although there was no statistically significant difference between these levels [52]. Therefore, we speculated that PN might cause PNALD, at least in part, by damaging the respiratory chain complex I assembly.

Insulin resistance is one of the metabolic phenotypes found in nonalcoholic fatty liver disease but there is limited clinical evidence for insulin resistance associated with PNALD. One report in piglets had demonstrated that chronic TPN induces significant insulin resistance [29]. In agreement with the previous study, our results showed upregulated NNMT, which correlates positively with insulin resistance [53–55], and downregulated GNMT, deficiency of which is known to impair glucose tolerance and insulin sensitivity. Furthermore, KEGG analysis indicated the DEPs to be associated with the insulin signaling pathway and PI3K-Akt signaling pathway (Table 3). These results together suggested that insulin sensitivity was impaired in patients with PNALD. Insulin resistance was also implicated by mitochondrial dysfunction or mitochondrial oxidative stress [56]. Mitochondrial oxidative phosphorylation was hampered, proteins with antioxidant function, including PRDX6, RGN, PHGDH, ALAD, GOT1, and DDAH1, were downregulated in patients with PNALD, and production of reactive oxygen species was increased, eventually leading to oxidative stress. Consistent with these results, several studies have demonstrated the impaired capacity of antioxidants during TPN [57, 58], and indicated oxidative stress play an important role in the development of PNALD [59, 60].

**Table 3:** KEGG pathways of differentially expressed proteins

S. No.		KEGG ID	Pathway	Protein number		
				List	Up	Down
1	Energy metabolism	00190	Oxidative phosphorylation	11	1	10
2	Carbohydrate metabolism	01200	Carbon metabolism	8	2	6
		00030	Pentose phosphate pathway	4	0	4
		00040	Pentose and glucuronate interconversions	4	1	3
		00051	Fructose and mannose metabolism	4	1	3
		00010	Glycolysis / Gluconeogenesis	3	0	3
		00052	Galactose metabolism	2	1	1
		00520	Amino sugar and nucleotide sugar metabolism	2	1	1
		00620	Pyruvate metabolism	2	1	1
		00630	Glyoxylate and dicarboxylate metabolism	2	1	1
3	Amino acid metabolism	01230	Biosynthesis of amino acids	5	1	4
		00260	Glycine, serin vitamin e and threonine metabolism	5	1	4
		00340	Histidine metabolism	5	0	5
		00350	Tyrosine metabolism	3	0	3
		00250	Alanine, aspartate and glutamate metabolism	3	1	2
		00270	Cysteine and methionine metabolism	3	0	3
		00220	Arginine biosynthesis	2	0	2
4	Lipid metabolism	00590	Arachidonic acid metabolism	2	0	2
		00561	Glycerolipid metabolism	2	1	1
		00140	Steroid hormone biosynthesis	2	0	2
5	Metabolism of cofactors and vitamins	00790	Folate biosynthesis	4	1	3
		00760	Nicotinate and nicotinamide metabolism	2	1	1
		00830	Retinol metabolism	2	0	2
6	Xenobiotics biodegradation and metabolism	00982	Drug metabolism - cytochrome P450	3	1	2
		00980	Metabolism of xenobiotics by cytochrome P450	3	0	3
		00983	Drug metabolism - other enzymes	2	0	2
7	Nucleotide metabolism	00240	Pyrimidine metabolism	2	0	2
8	Signal transduction	04020	Calcium signaling pathway	5	1	4
		04152	AMPK signaling pathway	3	0	2
		04151	PI3K-Akt signaling pathway	2	1	1
9	Nervous system	04723	Retrograde endocannabinoid signaling	10	0	10
10	Neurodegenerative diseases	05012	Parkinson’s disease	11	1	10
		05016	Huntington’s disease	11	1	10
		05010	Alzheimer’s disease	10	0	10
11	Endocrine system	04910	Insulin signaling pathway	6	0	6
		04922	Glucagon signaling pathway	5	0	5
12	Endocrine and metabolic diseases	04932	Non-alcoholic fatty liver disease (NAFLD)	10	0	10
13	Transport and catabolism	04142	Lysosome	5	5	0
		04144	Endocytosis	2	0	2
		04145	Phagosome	2	2	0
14	Cancers	05204	Chemical carcinogenesis	3	0	3
		05205	Proteoglycans in cancer	2	1	1
		05200	Pathways in cancer	2	0	2
15	Cell growth and death	04114	Oocyte meiosis	2	1	1
		04110	Cell cycle	2	1	1
		04217	Necroptosis	2	2	0
16	Cell motility	04810	Regulation of actin cytoskeleton	2	1	1
17	Immune system	04612	Antigen processing and presentation	2	2	0
		04621	NOD-like receptor signaling pathway	2	2	0
		04610	Complement and coagulation cascades	2	2	0
18	Infectious diseases	05120	Epithelial cell signaling in Helicobacter pylori infection	2	1	1
19	Digestive system	04976	Bile secretion	2	0	2
20	Excretory system	04966	Collecting duct acid secretion	2	1	1

Our results indicated that LGALS3 (**Table S**1), which contributes to inflammatory injury and fibrogenesis in cholestatic liver injury [61], was upregulated, and that DEPs were associated with NOD-like receptor signaling pathway. IPA results showed that the “LPS/IL-1 mediated inhibition of RXR function” pathway was activated **(Fig. S****2****).** Inflammation triggered by macrophages induced obesity related insulin resistance [62]. Consistent with these results, previous studies had indicated that LPS-mediated macrophages and IL-1β production or LPS-activated Kupffer cell through TLR4 might be early events in the pathogenesis of PNALD [63, 64].

A limitation of this study was an insufficient number of subjects, due to the extreme rarity of PNALD and infrequent source of liver tissue. Although previous studies had reported the abnormal glycolipid metabolism and oxidative stress in TPN, this is the first study that revealed damaged mitochondrial oxidative phosphorylation in patients with PNALD and provided a new target for exploring the mechanism and treatment of PNALD.

In conclusion, we identified several biological abnormalities that occur in patients with PNALD, such as in glycolipid metabolism, mitochondrial oxidative phosphorylation, and oxidative stress. We also detected the involvement of insulin and inflammatory signaling in PNALD pathogenesis. Our results provided new insights into the changes that occur in PNALD, and further identified candidate proteins as future PNALD biomarkers or therapeutic targets.

## Supplementary information

**Additional file 1. Supplementary information:** Supplementary tables and other bioinformation analysis (Annotation Enrichment Analysis, Different Proteins Analysis, Hierarchical Clustering Analysis, KEGG Analysis and, Network Analysis and Ingenuity Pathway Analysis) were listed in the file.

## Data Availability

All data generated or analyzed during this study are included in this published article (and its supplementary information files).

## Abbreviations

PN: Parenteral nutrition
PNALD: Parenteral nutrition-associated liver disease
iTRAQ: Isobaric Tag for Relative and Absolute Quantitation
GO: Gene Ontology
KEGG: Kyoto Encyclopedia of Genes and Genomes
DEPs: Differentially expressed proteins
TPN: Total parenteral nutrition
MF: Molecular functions
BP: Biological processes
CC: Cellular components
ACACB: Acetyl-CoA carboxylase 2
AKR1B1: Aldose reductase
ALAD: Delta-aminolevulinic acid dehydratase
AMDHD1: Probable imidazolonepropionase
CAPG: Macrophage-capping protein
CPPED1: Serine/threonine-protein phosphatase
CTSS: Cathepsin S
CYP2B6: Cytochrome P450 2B6
N(G)-dimethylarginine: N(G)
DDAH1: Dimethylaminohydrolase 1
EIF2: Eukaryotic translation initiation factor 2 subunit 1
FABP5: Fatty acid-binding protein
FBP1: Fructose-1,6-bisphosphatase 1
FBP2: Fructose-1,6-bisphosphatase isozyme 2
FTCD: Formimidoyltransferase-cyclodeaminase
GNMT: Glycine N-methyltransferase
GOT1: Aspartate aminotransferase
GOT1: Aspartate aminotransferase cytoplasmic
HAL: Histidine ammonia-lyase
HNMT: Histamine N-methyltransferase
IL-1β: Interleukin-1β
iTRAQ: Relative and Absolute Quantitation
IPA: Ingenuity Pathway Analysis
LGALS3: Galectin-3
LPS: Lipopolysaccharide
MF: Molecular function
NRF2: Nuclear factor erythroid 2-related factor 2 9.
NDUFA1: NADH dehydrogenase [ubiquinone] 1 alpha subcomplex subunit 1
NDUFA12: NADH dehydrogenase [ubiquinone] 1 alpha subcomplex subunit 12
NDUFA7: NADH dehydrogenase [ubiquinone] 1 alpha subcomplex subunit 7
NDUFB11: NADH dehydrogenase [ubiquinone] 1 beta subcomplex subunit 11
NDUFB7: NADH dehydrogenase [ubiquinone] 1 beta subcomplex subunit 7
NDUFS1: NADH-ubiquinone oxidoreductase 75 kDa subunit mitochondrial
NDUFS2: NADH dehydrogenase [ubiquinone] iron-sulfur protein 2
NDUFS8: NADH dehydrogenase [ubiquinone] iron-sulfur protein 8
NDUFV1: NADH dehydrogenase [ubiquinone] flavoprotein 1
NDUFV2: NADH dehydrogenase [ubiquinone] flavoprotein 2 mitochondrial
NNMT: Nicotinamide N-methyltransferase
PFKFB4: 6-phosphofructo-2-kinase/fructose-2,6-bisphosphatase 4
PC: Pyruvate carboxylase
PHGDH: D-3-phosphoglycerate dehydrogenase
PHKA2: Phosphorylase b kinase regulatory subunit alpha liver isoform
PHKB: Phosphorylase b kinase regulatory subunit beta
PHKG2: Phosphorylase b kinase gamma catalytic chain liver/testis isoform
PRDX6: Peroxiredoxin-6
RGN: Regucalcin
ROS: Reactive oxygen species
SULT1E1: Estrogen sulfotransferase
TLR4: Toll-like receptor 4
TPN: Total parenteral nutrition
UROC1: Urocanate hydratase
