# Supplementary material for: Proteome characteristics of liver tissue from patients with parenteral nutrition-associated liver disease
Source: Nutr Metab (Lond). 2020 Jun 3;17:43. doi: 10.1186/s12986-020-00453-z (PMC7268697; doi:10.1186/s12986-020-00453-z)
Supplement: Supplementary file 1 — Additional file 1. Supplementary information: Supplementary tables and other bioinformation analysis (Annotation Enrichment Analysis, Different Proteins Analysis, Hierarchical Clustering Analysis, KEGG Analysis and, Network Analysis and Ingenuity Pathway Analysis) were listed in the file. [file 12986_2020_453_MOESM1_ESM.docx]

Supplemental Information

Proteome Characteristics of Liver Tissue from Patients with Parenteral Nutrition-Associated Liver Disease

# Gulsudum Mamtawla^1^*; Feng Tian^1^*; Haifeng Sun^1^*; Li Zhang^1^; Xuejin Gao^1^; Bin Xue^2#^; Xinying Wang^1#^

| Table S1. Differentially expressed proteins in PNALD compared to control | | | | | | | | |
| --- | --- | --- | --- | --- | --- | --- | --- | --- |
| Accession | GENE name | Score | Coverage | Unique Peptides | PSMs | Fold changed (PNALD/Control) | p value | Expression |
| Q9UBS3 | DNAJB9 | 111.05 | 19.73 | 4 | 8 | 0.38041394 | 0.02121491 | DR |
| P20813 | CYP2B6 | 25.39 | 3.87 | 1 | 3 | 0.49485524 | 0.00997128 | DR |
| O94760 | DDAH1 | 565.06 | 45.26 | 12 | 40 | 0.56713526 | 0.00186212 | DR |
| O15239 | NDUFA1 | 30.15 | 8.57 | 1 | 1 | 0.59539597 | 0.00705084 | DR |
| Q9H8P0 | SRD5A3 | 24.49 | 2.2 | 1 | 2 | 0.60148357 | 0.00472016 | DR |
| Q93100 | PHKB | 20.56 | 0.64 | 1 | 1 | 0.62101354 | 0.0120043 | DR |
| Q9UBR1 | UPB1 | 1215.28 | 42.71 | 15 | 55 | 0.63143308 | 0.0316059 | DR |
| O43704 | SULT1B1 | 25.57 | 3.04 | 1 | 2 | 0.65341447 | 0.01626344 | DR |
| Q15024 | EXOSC7 | 35.77 | 5.15 | 1 | 1 | 0.65582432 | 0.04528561 | DR |
| Q9UI09 | NDUFA12 | 131.19 | 13.79 | 2 | 10 | 0.66287795 | 0.00490436 | DR |
| O00757 | FBP2 | 176.78 | 8.26 | 1 | 13 | 0.67448433 | 0.01772031 | DR |
| P09327 | VIL1 | 151.22 | 14.51 | 11 | 21 | 0.6771159 | 0.01464719 | DR |
| P00326 | ADH1C | 38182.83 | 36.8 | 3 | 1144 | 0.67769671 | 0.01637438 | DR |
| P50225 | SULT1A1 | 207.50 | 31.53 | 4 | 47 | 0.68136837 | 0.04291248 | DR |
| O95954 | FTCD | 1412.10 | 34.94 | 16 | 66 | 0.68179413 | 0.03567242 | DR |
| Q9NX14 | NDUFB11 | 0.00 | 4.58 | 1 | 2 | 0.69931058 | 0.01206567 | DR |
| P15735 | PHKG2 | 32.18 | 4.19 | 2 | 2 | 0.70280812 | 0.04159222 | DR |
| P61457 | PCBD1 | 222.41 | 51.92 | 4 | 21 | 0.70301844 | 0.0227637 | DR |
| Q9NZN3 | EHD3 | 209.63 | 19.25 | 4 | 18 | 0.70555574 | 0.00490329 | DR |
| O00217 | NDUFS8 | 27.24 | 5.24 | 1 | 2 | 0.70904213 | 0.00411531 | DR |
| O75191 | XYLB | 85.36 | 6.72 | 3 | 6 | 0.71129476 | 0.00616395 | DR |
| Q9UN36 | NDRG2 | 154.38 | 32.35 | 10 | 21 | 0.7124086 | 0.02823052 | DR |
| Q6XQN6 | NAPRT | 920.32 | 33.09 | 13 | 43 | 0.72132614 | 0.0480321 | DR |
| Q14749 | GNMT | 355.60 | 34.92 | 8 | 18 | 0.72197212 | 0.02670997 | DR |
| Q8N8N7 | PTGR2 | 93.79 | 7.41 | 2 | 4 | 0.7223441 | 0.00607797 | DR |
| P62760 | VSNL1 | 53.93 | 17.8 | 3 | 6 | 0.72347158 | 0.04948653 | DR |
| O75884 | RBBP9 | 42.01 | 4.3 | 1 | 2 | 0.72427986 | 0.03348102 | DR |
| Q8IVS8 | GLYCTK | 105.49 | 7.27 | 4 | 6 | 0.72517473 | 0.00259268 | DR |
| O95182 | NDUFA7 | 100.60 | 16.81 | 2 | 8 | 0.73217563 | 0.04704381 | DR |
| P49888 | SULT1E1 | 28.44 | 9.52 | 3 | 4 | 0.73224401 | 0.01475923 | DR |
| P13716 | ALAD | 838.78 | 32.12 | 7 | 32 | 0.74090989 | 0.02312581 | DR |
| P09467 | FBP1 | 1813.06 | 52.66 | 14 | 132 | 0.74190198 | 0.0020621 | DR |
| P32320 | CDA | 25.19 | 4.79 | 1 | 2 | 0.74503768 | 0.01581913 | DR |
| Q96N76 | UROC1 | 272.36 | 22.78 | 14 | 28 | 0.74875902 | 0.03903793 | DR |
| P49821 | NDUFV1 | 120.77 | 7.97 | 4 | 9 | 0.75032532 | 0.02002849 | DR |
| P30047 | GCHFR | 240.24 | 48.81 | 2 | 17 | 0.75218596 | 0.04025216 | DR |
| P46019 | PHKA2 | 26.02 | 3.64 | 3 | 4 | 0.75691761 | 0.0179831 | DR |
| O43175 | PHGDH | 610.31 | 22.14 | 12 | 40 | 0.75963096 | 0.0081446 | DR |
| P32929 | CTH | 263.65 | 32.35 | 11 | 33 | 0.76570998 | 0.04357269 | DR |
| Q16630 | CPSF6 | 48.70 | 2.54 | 1 | 3 | 0.76585154 | 0.00703521 | DR |
| Accession | GENE name | Score | Coverage | Unique Peptides | PSMs | Fold changed (PNALD/Control) | p value | Expression |
| O75306 | NDUFS2 | 184.43 | 8.64 | 3 | 14 | 0.76897109 | 0.02522765 | DR |
| P46952 | HAAO | 905.54 | 64.34 | 14 | 42 | 0.77191615 | 0.02354827 | DR |
| P00918 | CA2 | 440.24 | 45.77 | 10 | 43 | 0.77303708 | 0.01751267 | DR |
| P42357 | HAL | 115.12 | 18.42 | 11 | 14 | 0.77361893 | 0.03503982 | DR |
| Q5T5P2 | KIAA1217 | 19.39 | 1.13 | 2 | 2 | 0.77675372 | 0.0063313 | DR |
| O00763 | ACACB | 178.47 | 6.55 | 15 | 24 | 0.7776345 | 0.04896584 | DR |
| P00533 | EGFR | 43.49 | 1.57 | 2 | 2 | 0.77795589 | 0.00970475 | DR |
| O15260 | SURF4 | 1117.40 | 10.04 | 2 | 32 | 0.78178055 | 0.03297981 | DR |
| P28331 | NDUFS1 | 155.45 | 16.09 | 10 | 22 | 0.7864899 | 0.01001448 | DR |
| Q15493 | RGN | 624.10 | 27.76 | 8 | 34 | 0.79042973 | 0.04911236 | DR |
| Q96NU7 | AMDHD1 | 422.04 | 23 | 7 | 23 | 0.79088824 | 0.02485017 | DR |
| Q9ULA0 | DNPEP | 284.66 | 21.26 | 7 | 14 | 0.79425269 | 0.02112214 | DR |
| P17174 | GOT1 | 711.76 | 59.56 | 22 | 83 | 0.79430356 | 0.01626353 | DR |
| Q93099 | HGD | 479.64 | 31.91 | 12 | 33 | 0.79442739 | 0.03949338 | DR |
| P50135 | HNMT | 143.37 | 20.55 | 7 | 11 | 0.79542305 | 0.03786244 | DR |
| Q14376 | GALE | 373.83 | 41.95 | 11 | 20 | 0.79568164 | 0.03712683 | DR |
| P63208 | SKP1 | 48.64 | 11.04 | 1 | 2 | 0.79608197 | 0.01918288 | DR |
| P04424 | ASL | 853.85 | 48.49 | 21 | 91 | 0.79752729 | 0.0168237 | DR |
| Q9BRF8 | CPPED1 | 422.73 | 25.8 | 7 | 20 | 0.79832289 | 0.03997091 | DR |
| Q9BUT1 | BDH2 | 75.64 | 17.96 | 5 | 11 | 0.80040907 | 0.04087896 | DR |
| Q9NQX3 | GPHN | 108.69 | 9.24 | 5 | 5 | 0.80069098 | 0.03381524 | DR |
| P52294 | KPNA1 | 25.99 | 3.35 | 1 | 1 | 0.80447472 | 0.02039613 | DR |
| P17568 | NDUFB7 | 70.13 | 18.25 | 2 | 5 | 0.80709165 | 0.00197637 | DR |
| Q27J81 | INF2 | 23.49 | 1.44 | 2 | 2 | 0.81333627 | 0.01220438 | DR |
| Q00796 | SORD | 2006.89 | 40.62 | 13 | 109 | 0.81364553 | 0.04332684 | DR |
| P49006 | MARCKSL1 | 42.93 | 14.36 | 2 | 2 | 0.81820931 | 0.01020595 | DR |
| P16152 | CBR1 | 1142.01 | 64.26 | 13 | 65 | 0.82008042 | 0.03141233 | DR |
| P23526 | AHCY | 979.51 | 40.05 | 15 | 77 | 0.82018485 | 0.00705234 | DR |
| Q9Y6L6 | SLCO1B1 | 73.62 | 9.55 | 4 | 13 | 0.82074067 | 0.01282468 | DR |
| P30041 | PRDX6 | 743.46 | 58.04 | 11 | 61 | 0.82444468 | 0.02013316 | DR |
| Q9Y2S2 | CRYL1 | 743.25 | 38.87 | 12 | 46 | 0.82457911 | 0.04521075 | DR |
| P19404 | NDUFV2 | 38.37 | 13.65 | 4 | 4 | 0.82482872 | 0.03999595 | DR |
| Q96K76 | USP47 | 93.95 | 2.4 | 3 | 4 | 0.82661105 | 0.04114926 | DR |
| Q14847 | LASP1 | 323.65 | 34.87 | 8 | 15 | 1.2018859 | 0.04764529 | UR |
| Q04917 | YWHAH | 310.89 | 35.77 | 5 | 27 | 1.21392902 | 0.0125373 | UR |
| O00429 | DNM1L | 121.32 | 6.11 | 3 | 9 | 1.21468633 | 0.03181989 | UR |
| P05155 | SERPING1 | 76.38 | 12 | 5 | 7 | 1.22456519 | 0.04531059 | UR |
| P05154 | SERPINA5 | 41.43 | 4.19 | 1 | 3 | 1.24439305 | 0.02634528 | UR |
| P19397 | CD53 | 35.16 | 3.65 | 1 | 1 | 1.24808005 | 0.00157567 | UR |
| P30419 | NMT1 | 150.44 | 6.45 | 2 | 8 | 1.25156738 | 0.02496583 | UR |
| P45880 | VDAC2 | 222.65 | 13.95 | 4 | 16 | 1.25493359 | 0.04461711 | UR |
| P21281 | ATP6V1B2 | 62.34 | 10.18 | 4 | 5 | 1.25720144 | 0.03498476 | UR |
| Accession | GENE name | Score | Coverage | Unique Peptides | PSMs | Fold changed (PNALD/Control) | p value | Expression |
| O00330 | PDHX | 22.62 | 2 | 1 | 2 | 1.26907217 | 0.00762845 | UR |
| P63313 | TMSB10 | 36.00 | 45.45 | 1 | 4 | 1.28780527 | 0.02079599 | UR |
| Q99729 | HNRNPAB | 44.21 | 9.64 | 2 | 4 | 1.29116936 | 0.04859804 | UR |
| Q00325 | SLC25A3 | 335.32 | 28.18 | 11 | 32 | 1.29732984 | 0.0342526 | UR |
| Q9H299 | SH3BGRL3 | 143.03 | 47.31 | 5 | 10 | 1.30875445 | 0.04943518 | UR |
| O75323 | NIPSNAP2 | 23.61 | 6.64 | 1 | 6 | 1.31289278 | 0.04127471 | UR |
| Q9Y6N5 | SQOR | 213.52 | 23.56 | 9 | 19 | 1.33134502 | 0.04280337 | UR |
| Q5NE16 | CTSL3P | 29.62 | 3.21 | 1 | 3 | 1.33604647 | 0.00068497 | UR |
| P10619 | CTSA | 39.34 | 2.71 | 1 | 2 | 1.33955828 | 0.00915066 | UR |
| P61225 | RAP2B | 132.73 | 26.23 | 1 | 6 | 1.34614472 | 0.03029339 | UR |
| Q14764 | MVP | 44.13 | 5.82 | 5 | 6 | 1.3517368 | 0.00916586 | UR |
| P11498 | PC | 2265.29 | 43.72 | 43 | 143 | 1.37988691 | 0.00114998 | UR |
| O00244 | ATOX1 | 35.29 | 23.53 | 2 | 3 | 1.4038282 | 0.00600902 | UR |
| P22087 | FBL | 82.58 | 6.23 | 1 | 1 | 1.41029441 | 0.03675124 | UR |
| P26038 | MSN | 1374.98 | 49.57 | 25 | 100 | 1.42245528 | 0.03625741 | UR |
| P31513 | FMO3 | 708.95 | 35.34 | 17 | 75 | 1.43329277 | 0.01934539 | UR |
| P07686 | HEXB | 71.49 | 11.87 | 6 | 12 | 1.43722693 | 0.00100326 | UR |
| Q9HAV7 | GRPEL1 | 44.87 | 8.76 | 2 | 6 | 1.45206289 | 0.03705973 | UR |
| P40261 | NNMT | 276.57 | 19.32 | 5 | 17 | 1.48135344 | 0.0190958 | UR |
| P21549 | AGXT | 3075.67 | 48.47 | 16 | 156 | 1.49037687 | 0.02387458 | UR |
| P07602 | PSAP | 204.40 | 6.68 | 4 | 18 | 1.5137847 | 0.03263189 | UR |
| Q9NUV9 | GIMAP4 | 200.34 | 7.9 | 2 | 7 | 1.51559698 | 0.02605651 | UR |
| P15121 | AKR1B1 | 39.20 | 8.23 | 2 | 6 | 1.52108362 | 0.02538253 | UR |
| Q99538 | LGMN | 271.05 | 6.93 | 3 | 12 | 1.55404581 | 0.00394202 | UR |
| P01011 | SERPINA3 | 250.93 | 19.62 | 6 | 21 | 1.56965592 | 0.00108599 | UR |
| Q01469 | FABP5 | 64.69 | 18.52 | 3 | 3 | 1.65886456 | 0.00334758 | UR |
| P25774 | CTSS | 38.85 | 12.39 | 4 | 5 | 1.85422845 | 0.00336907 | UR |
| P40121 | CAPG | 77.19 | 7.47 | 2 | 4 | 1.97219971 | 0.02185637 | UR |
| P17931 | LGALS3 | 30.90 | 7.2 | 2 | 5 | 2.05429049 | 0.00037386 | UR |
| Q14956 | GPNMB | 73.73 | 1.57 | 1 | 4 | 4.08983471 | 0.01932368 | UR |
| Score: the sum of the scores of the individual peptides; Coverage: default the percentage of the protein sequence covered by  identified peptides; Unique Peptides: the number of peptide sequences unique to a protein group; PSMs: The total number of  identified peptide sequences (peptide spectrum matches) for the protein, including those redundantly identified; DR, down regulated; UR, up regulated. | | | | | | | | |

| Table.S2 Mitochondrial and mitochondrial-related differentially expressed proteins | | | | | | | | |
| --- | --- | --- | --- | --- | --- | --- | --- | --- |
| Accession | GENE name | Score | Coverage | Unique Peptides | PSMs | Fold changed (PNALD/Control) | p value | Expression |
| O94760 | DDAH1 | 565.06306 | 45.26 | 12 | 40 | 0.56713526 | 0.00186212 | DR |
| P19404 | NDUFV2 | 38.37 | 13.65 | 4 | 4 | 0.82482872 | 0.03999595 | DR |
| P17568 | NDUFB7 | 70.12671 | 18.25 | 2 | 5 | 0.80709165 | 0.00197637 | DR |
| P28331 | NDUFS1 | 155.45046 | 16.09 | 10 | 22 | 0.7864899 | 0.01001448 | DR |
| P49821 | NDUFV1 | 120.77195 | 7.97 | 4 | 9 | 0.75032532 | 0.02002849 | DR |
| O75306 | NDUFS2 | 184.43441 | 8.64 | 3 | 14 | 0.76897109 | 0.02522765 | DR |
| O95182 | NDUFA7 | 100.60443 | 16.81 | 2 | 8 | 0.73217563 | 0.04704381 | DR |
| O15239 | NDUFA1 | 30.15 | 8.57 | 1 | 1 | 0.59539597 | 0.00705084 | DR |
| Q9UI09 | NDUFA12 | 131.18737 | 13.79 | 2 | 10 | 0.66287795 | 0.00490436 | DR |
| Q9NX14 | NDUFB11 | 0 | 4.58 | 1 | 2 | 0.69931058 | 0.01206567 | DR |
| O00217 | NDUFS8 | 27.24 | 5.24 | 1 | 2 | 0.70904213 | 0.00411531 | DR |
| Q8IVS8 | GLYCTK | 105.49058 | 7.27 | 4 | 6 | 0.72517473 | 0.00259268 | DR |
| O00763 | ACACB | 178.46903 | 6.55 | 15 | 24 | 0.7776345 | 0.04896584 | DR |
| P17174 | GOT1 | 711.75806 | 59.56 | 22 | 83 | 0.79430356 | 0.01626353 | DR |
| Q9BUT1 | BDH2 | 75.63749 | 17.96 | 5 | 11 | 0.80040907 | 0.04087896 | DR |
| Q00796 | SORD | 2006.88873 | 40.62 | 13 | 109 | 0.81364553 | 0.04332684 | DR |
| Q04917 | YWHAH | 310.89152 | 35.77 | 5 | 27 | 1.21392902 | 0.0125373 | UR |
| O00429 | DNM1L | 121.31924 | 6.11 | 3 | 9 | 1.21468633 | 0.03181989 | UR |
| P30419 | NMT1 | 150.43951 | 6.45 | 2 | 8 | 1.25156738 | 0.02496583 | UR |
| P45880 | VDAC2 | 222.64677 | 13.95 | 4 | 16 | 1.25493359 | 0.04461711 | UR |
| O00330 | PDHX | 22.62 | 2 | 1 | 2 | 1.26907217 | 0.00762845 | UR |
| Q00325 | SLC25A3 | 335.31673 | 28.18 | 11 | 32 | 1.29732984 | 0.0342526 | UR |
| P11498 | PC | 2265.29216 | 43.72 | 43 | 143 | 1.37988691 | 0.00114998 | UR |
| Q9HAV7 | GRPEL1 | 44.869 | 8.76 | 2 | 6 | 1.45206289 | 0.03705973 | UR |
| P21549 | AGXT | 3075.67009 | 48.47 | 16 | 156 | 1.49037687 | 0.02387458 | UR |
| P07602 | PSAP | 204.40321 | 6.68 | 4 | 18 | 1.5137847 | 0.03263189 | UR |
| P17931 | LGALS3 | 30.89819 | 7.2 | 2 | 5 | 2.05429049 | 0.00037386 | UR |
| Score: the sum of the scores of the individual peptides; Coverage: default the percentage of the protein sequence covered by identified peptides; Unique Peptides: the number of peptide sequences unique to a protein group; PSMs: The total number of identified peptide sequences (peptide spectrum matches) for the protein, including those redundantly identified; DR, down regulated; UR, up regulated. | | | | | | | | |

| Table.S3 Glycolipid metabolisms associated differentially expressed proteins | | | | | | | | |
| --- | --- | --- | --- | --- | --- | --- | --- | --- |
| Accession | GENE name | Score | Coverage | Unique  Peptides | PSMs | Fold changed (PNALD/Control) | p value | Expression |
| O15239 | NDUFA1 | 30.15 | 8.57 | 1 | 1 | 0.59539597 | 0.00705084 | DR |
| Q93100 | PHKB | 20.56 | 0.64 | 1 | 1 | 0.62101354 | 0.0120043 | DR |
| Q9UI09 | NDUFA12 | 131.18737 | 13.79 | 2 | 10 | 0.66287795 | 0.00490436 | DR |
| O00757 | FBP2 | 176.78375 | 8.26 | 1 | 13 | 0.67448433 | 0.01772031 | DR |
| P00326 | ADH1C | 38182.8308 | 36.8 | 3 | 1144 | 0.67769671 | 0.01637438 | DR |
| Q9NX14 | NDUFB11 | 0 | 4.58 | 1 | 2 | 0.69931058 | 0.01206567 | DR |
| P15735 | PHKG2 | 32.18 | 4.19 | 2 | 2 | 0.70280812 | 0.04159222 | DR |
| P61457 | PCBD1 | 222.40797 | 51.92 | 4 | 21 | 0.70301844 | 0.0227637 | DR |
| O00217 | NDUFS8 | 27.24 | 5.24 | 1 | 2 | 0.70904213 | 0.00411531 | DR |
| O75191 | XYLB | 85.36059 | 6.72 | 3 | 6 | 0.71129476 | 0.00616395 | DR |
| Q8IVS8 | GLYCTK | 105.49058 | 7.27 | 4 | 6 | 0.72517473 | 0.00259268 | DR |
| O95182 | NDUFA7 | 100.60443 | 16.81 | 2 | 8 | 0.73217563 | 0.04704381 | DR |
| P09467 | FBP1 | 1813.05616 | 52.66 | 14 | 132 | 0.74190198 | 0.0020621 | DR |
| P49821 | NDUFV1 | 120.77195 | 7.97 | 4 | 9 | 0.75032532 | 0.02002849 | DR |
| P46019 | PHKA2 | 26.01551 | 3.64 | 3 | 4 | 0.75691761 | 0.0179831 | DR |
| O43175 | PHGDH | 610.30877 | 22.14 | 12 | 40 | 0.75963096 | 0.0081446 | DR |
| O75306 | NDUFS2 | 184.43441 | 8.64 | 3 | 14 | 0.76897109 | 0.02522765 | DR |
| O00763 | ACACB | 178.46903 | 6.55 | 15 | 24 | 0.7776345 | 0.04896584 | DR |
| P28331 | NDUFS1 | 155.45046 | 16.09 | 10 | 22 | 0.7864899 | 0.01001448 | DR |
| Q15493 | RGN | 624.09647 | 27.76 | 8 | 34 | 0.79042973 | 0.04911236 | DR |
| P17174 | GOT1 | 711.75806 | 59.56 | 22 | 83 | 0.79430356 | 0.01626353 | DR |
| Q14376 | GALE | 373.8254 | 41.95 | 11 | 20 | 0.79568164 | 0.03712683 | DR |
| Q9NQX3 | GPHN | 108.68857 | 9.24 | 5 | 5 | 0.80069098 | 0.03381524 | DR |
| P17568 | NDUFB7 | 70.12671 | 18.25 | 2 | 5 | 0.80709165 | 0.00197637 | DR |
| Q00796 | SORD | 2006.88873 | 40.62 | 13 | 109 | 0.81364553 | 0.04332684 | DR |
| P16152 | CBR1 | 1142.00898 | 64.26 | 13 | 65 | 0.82008042 | 0.03141233 | DR |
| Q9Y2S2 | CRYL1 | 743.24886 | 38.87 | 12 | 46 | 0.82457911 | 0.04521075 | DR |
| P19404 | NDUFV2 | 38.37 | 13.65 | 4 | 4 | 0.82482872 | 0.03999595 | DR |
| P21281 | ATP6V1B2 | 62.3425 | 10.18 | 4 | 5 | 1.25720144 | 0.03498476 | UR |
| P11498 | PC | 2265.29216 | 43.72 | 43 | 143 | 1.37988691 | 0.00114998 | UR |
| P21549 | AGXT | 3075.67009 | 48.47 | 16 | 156 | 1.49037687 | 0.02387458 | UR |
| P15121 | AKR1B1 | 39.1969 | 8.23 | 2 | 6 | 1.52108362 | 0.02538253 | UR |
| Score: the sum of the scores of the individual peptides; Coverage: default the percentage of the protein sequence covered by identified peptides; Unique Peptides: the number of peptide sequences unique to a protein group; PSMs: The total number of identified peptide sequences (peptide spectrum matches) for the protein, including those redundantly identified; DR, down regulated; UR, up regulated. | | | | | | | | |

| Table.S4 Amino acid metabolisms associated differentially expressed proteins | | | | | | | | |
| --- | --- | --- | --- | --- | --- | --- | --- | --- |
| Accession | Gene name | Score | Coverage | Unique  Peptides | PSMs | Fold changed (PNALD/Control) | p value | Expression |
| O94760 | DDAH1 | 565.06306 | 45.26 | 12 | 40 | 0.56713526 | 0.00186212 | DR |
| O95954 | FTCD | 1412.10439 | 34.94 | 16 | 66 | 0.68179413 | 0.03567242 | DR |
| P61457 | PCBD1 | 222.40797 | 51.92 | 4 | 21 | 0.70301844 | 0.0227637 | DR |
| Q14749 | GNMT | 355.59732 | 34.92 | 8 | 18 | 0.72197212 | 0.02670997 | DR |
| P09467 | FBP1 | 1813.05616 | 52.66 | 14 | 132 | 0.74190198 | 0.0020621 | DR |
| P32320 | CDA | 25.19 | 4.79 | 1 | 2 | 0.74503768 | 0.01581913 | DR |
| Q96N76 | UROC1 | 272.36232 | 22.78 | 14 | 28 | 0.74875902 | 0.03903793 | DR |
| P32929 | CTH | 263.64976 | 32.35 | 11 | 33 | 0.76570998 | 0.04357269 | DR |
| Q16630 | CPSF6 | 48.6991 | 2.54 | 1 | 3 | 0.76585154 | 0.00703521 | DR |
| P42357 | HAL | 115.12091 | 18.42 | 11 | 14 | 0.77361893 | 0.03503982 | DR |
| O00763 | ACACB | 178.46903 | 6.55 | 15 | 24 | 0.7776345 | 0.04896584 | DR |
| Q96NU7 | AMDHD1 | 422.04158 | 23 | 7 | 23 | 0.79088824 | 0.02485017 | DR |
| Q93099 | HGD | 479.63674 | 31.91 | 12 | 33 | 0.79442739 | 0.03949338 | DR |
| P50135 | HNMT | 143.37456 | 20.55 | 7 | 11 | 0.79542305 | 0.03786244 | DR |
| P04424 | ASL | 853.85488 | 48.49 | 21 | 91 | 0.79752729 | 0.0168237 | DR |
| O00429 | DNM1L | 121.31924 | 6.11 | 3 | 9 | 1.21468633 | 0.03181989 | UR |
| Q5NE16 | CTSL3P | 29.62 | 3.21 | 1 | 3 | 1.33604647 | 0.00068497 | UR |
| P10619 | CTSA | 39.34 | 2.71 | 1 | 2 | 1.33955828 | 0.00915066 | UR |
| P61225 | RAP2B | 132.72808 | 26.23 | 1 | 6 | 1.34614472 | 0.03029339 | UR |
| Q99538 | LGMN | 271.05054 | 6.93 | 3 | 12 | 1.55404581 | 0.00394202 | UR |
| P25774 | CTSS | 38.85367 | 12.39 | 4 | 5 | 1.85422845 | 0.00336907 | UR |
| Q14956 | GPNMB | 73.72934 | 1.57 | 1 | 4 | 4.08983471 | 0.01932368 | UR |
| Score: the sum of the scores of the individual peptides; Coverage: default the percentage of the protein sequence covered by  identified peptides; Unique Peptides: the number of peptide sequences unique to a protein group; PSMs: The total number of  identified peptide sequences (peptide spectrum matches) for the protein, including those redundantly identified; DR, down regulated; UR, up regulated. | | | | | | | | |

#### Supplementary Figures

**
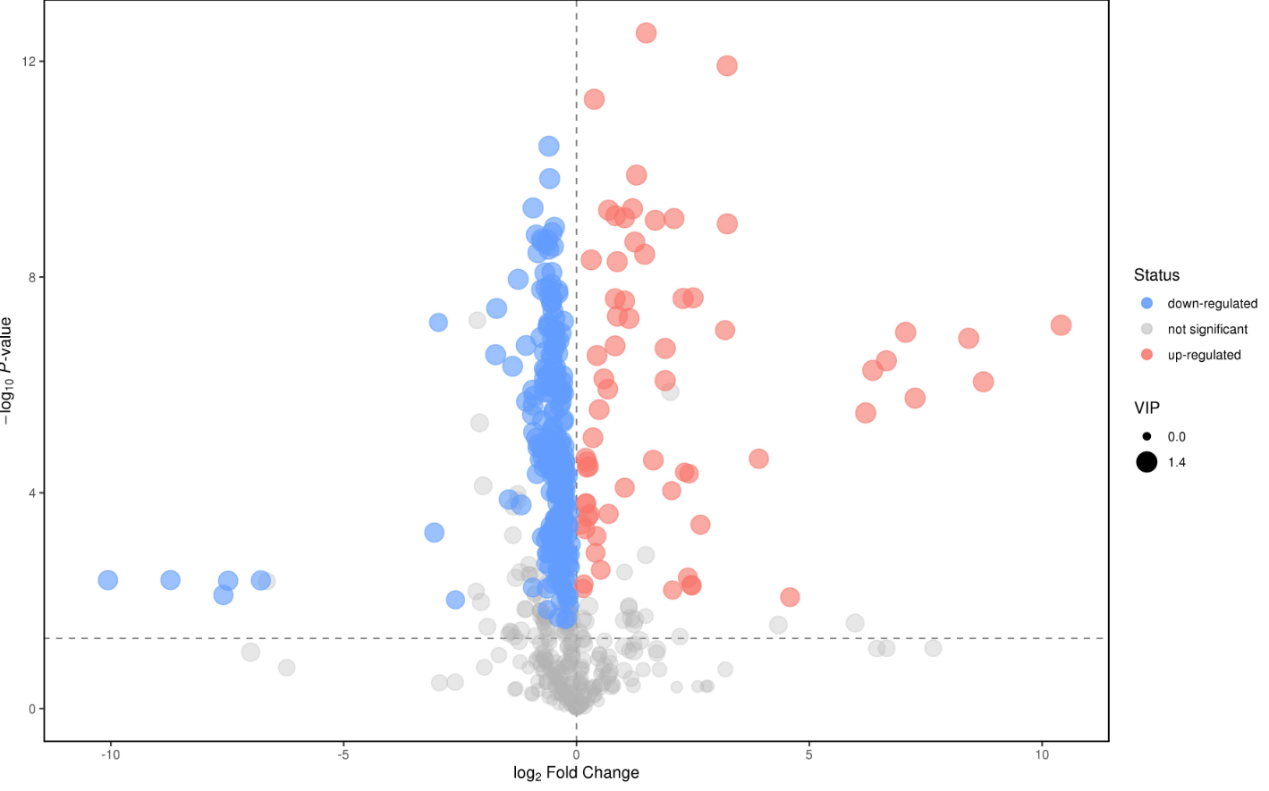
**

**Fig. S1.** Volcano plot of the quantified proteins (the proteins that showed significant down- and up-regulated after statistical analysis are reported in blue and red)


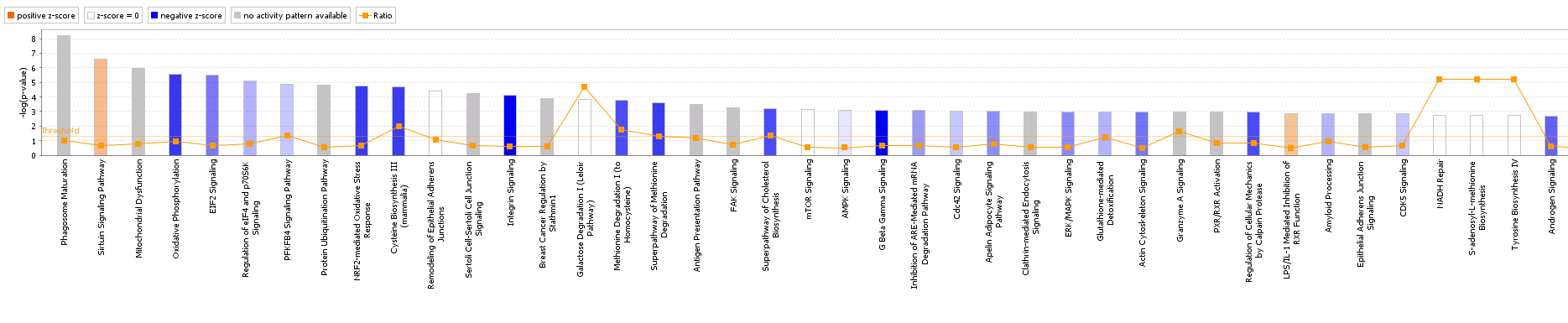


**Fig. S2.** The significant enrichment of differential proteins in the classical pathway was demonstrated. The abscissa is the name of the pathway, and the ordinate is the significance level of enrichment (negative logarithmic transformation with base 10). The orange label indicates that the pathway is activated (z-score > 0), the blue label indicates that the pathway is inhibited (z-score <0), the depth of orange and blue (or the absolute value of z-score) represents the degree of activation or inhibition. Oxidative phosphorylation was significantly inhibited with a z-score of -2.496


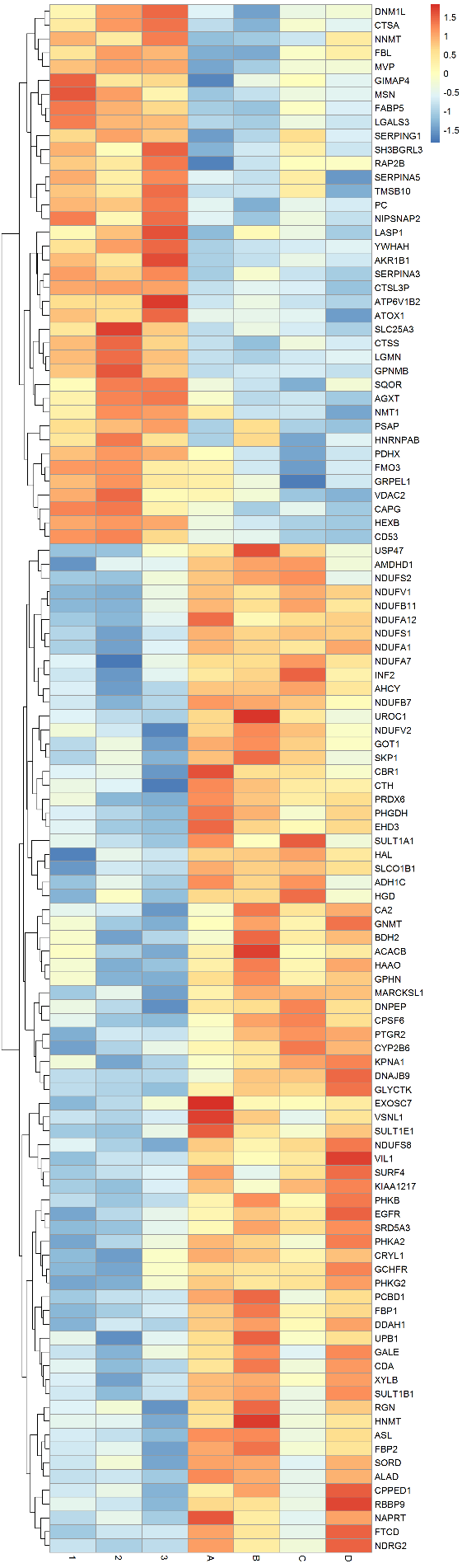


**Fig. S3.** Heat map of differentially expressed proteins (DEPs) in PNALD and control patient. Colored boxes represent up-regulation (red) and down-regulation (blue) in the PNALD. The color scale shown at the upper right indicates the fold changes in protein expression of all the samples. 1, 2, 3: PNALD group; A, B, C, D: Control group.
